# Microstructural white matter abnormalities in pediatric and adult obsessive‐compulsive disorder: A systematic review and meta‐analysis

**DOI:** 10.1002/brb3.1975

**Published:** 2020-12-03

**Authors:** Qian Li, Youjin Zhao, Zixuan Huang, Yi Guo, Jingyi Long, Lekai Luo, Wanfang You, John A. Sweeney, Fei Li, Qiyong Gong

**Affiliations:** ^1^ Huaxi MR Research Center (HMRRC), Department of Radiology West China Hospital of Sichuan University Chengdu China; ^2^ Research Unit of Psychoradiology Chinese Academy of Medical Sciences Chengdu China; ^3^ Functional and Molecular Imaging Key Laboratory of Sichuan Province, West China Hospital of Sichuan University Chengdu China; ^4^ Medical Imaging Technology Department, West China School of Medicine Sichuan University Chengdu China; ^5^ Department of Psychiatry University of Cincinnati Cincinnati OH USA

**Keywords:** Psychoradiology, fractional anisotropy, meta‐analysis, obsessive‐compulsive disorder, RRID:SCR_002823, RRID:SCR_002554, tract‐based spatial statistics, white matter microstructure

## Abstract

**Objective:**

To identify the most prominent and replicable fractional anisotropy (FA) alterations of white matter associated with obsessive‐compulsive disorder (OCD) in tract‐based spatial statistics (TBSS) studies.

**Methods:**

We reviewed previous TBSS studies (*n* = 20) in OCD and performed a meta‐analysis (*n* = 16) of FA differences.

**Results:**

No between‐group differences in FA were detected in the pooled meta‐analysis. However, reduced FA was identified in the genu and anterior body of corpus callosum (CC) in adult OCD. FA reductions in the anterior body of CC were associated with a later age of onset in adult patients with OCD. For pediatric OCD, decreased FA in earlier adolescence and increased FA in later adolescence were seemingly related to an altered trajectory of brain maturation.

**Conclusions:**

Absent in the pooled sample but robust in adults, disrupted microstructural organization in the anterior part of CC indicates a bias of deficits toward connections in interhemispheric connections of rostral neocortical regions, which could lead to deficits of interhemispheric communication and thus contribute to cognitive and emotional deficits in adult OCD. The correlation between FA in the anterior body of CC and older illness onset suggests that patients with later adult onset of illness may represent a biologically distinct subgroup. For pediatric OCD, alterations in neurodevelopmental maturation may contribute to inconsistent patterns of FA alteration relative to controls during adolescence. While most studies of OCD have emphasized alterations of within hemisphere fronto‐striatal circuits, these results indicate that between hemisphere connectivity of this circuitry may also represent important pathophysiology of the illness.

## Graphical abstract

No between‐group differences in FA were detected in the pooled meta‐analysis. However, reduced FA was identified in the genu and anterior body of corpus callosum (CC) in adult OCD. FA reductions in the anterior body of CC were associated with a later age of onset in adult patients with OCD. For pediatric OCD, decreased FA in earlier adolescence and increased FA in later adolescence is seemingly related to an altered trajectory of brain maturation.

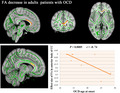

## 1 INTRODUCTION

Obsessive‐compulsive disorder (OCD) is characterized by unwanted, ego‐dystonic, recurrent and persistent thoughts (i.e., obsessions) and urges (i.e., compulsions), and has a lifetime population prevalence of approximately 2.3% (Ruscio et al., 2010). Despite its prevalence and associated morbidity, it has received relatively modest emphasis in psychiatric research programs. In part, for this reason, the pathophysiological mechanisms of OCD are not yet fully elucidated, though substantial progress has been achieved in recent years. Psychoradiology is an emerging subspecialty of radiology, and it's rapid development has led to our better understanding of the complex brain abnormalities in patients with psychiatric disorders (Gong, 2020). Dysfunction and structural deficits of cortico‐striato‐thalamo‐cortical (CSTC) circuits, especially fronto‐striatal circuits, have been proposed to play a central role in the pathogenesis of OCD (Aouizerate et al., 2004; Menzies et al., 2008). Previous meta‐ and mega‐analyses of magnetic resonance imaging (MRI) data have identified abnormalities of gray matter volumes (GMV) in fronto‐striatal circuits, including orbitofrontal gyrus (Rotge et al., 2010; de Wit et al., 2014), cingulate cortex (Eng et al., 2015; de Wit et al., 2014), striatum (caudate nucleus, putamen, and pallidum), and thalamus (Boedhoe et al., 2017; Eng et al., 2015), and have found hyperactivation/hypoactivation in prefrontal, cingulate, and striatal brain regions in OCD (Eng et al., 2015; Gursel et al., 2018). Structural changes in the hippocampus, dorsolateral prefronto‐striatal circuits, and in temporo‐parieto‐occipital associative brain regions have also been reported (Boedhoe et al., 2017, 2018; Piras et al., 2015). Widespread microstructural abnormalities of white matter (WM) tracts have been identified using diffusion tensor imaging (DTI) (Koch et al., 2014), consistent with gene association studies indicating an important role for WM abnormalities in the etiology of OCD (Bubb et al., 2018; Gassó et al., 2015).

Fractional anisotropy (FA), the most frequently reported index in DTI, measures the proportion of water diffusion in the primary direction (generally aligned with the dominant white matter bundle within a voxel) compared to other directions and thus reflects characteristics of WM microstructure (e.g., fiber packing, axonal diameter, thickness of myelin sheaths, and directionality of fibers) (Koch et al., 2014; Le Bihan et al., 2001). Analyzing FA of whole‐brain WM using voxel‐based analysis (VBA) and tract‐based spatial statistics (TBSS) can overcome limitations of region of interest (ROI) analysis. The TBSS method restricts analysis to the center of major WM tracts by projecting each subject's FA data onto the mean skeleton, reducing misalignment problems, and spatial smoothing bias that can arise from the use of the VBA method (Smith et al., 2006). Studies of OCD using TBSS have reported decreased FA in the corpus callosum (CC) (Gan et al., 2017; Zhou et al., 2018), corona radiata (Benedetti et al., 2013), superior longitudinal fasciculus (Spalletta et al., 2014), and internal capsule (Fontenelle et al., 2011). Other studies found no significant differences in FA between patients with OCD and healthy controls (HCs) (Ameis et al., 2016; Magioncalda et al., 2016). Some studies reported increased FA in the cerebellum (Hartmann et al., 2016), inferior frontal‐occipital fasciculus, inferior longitudinal fasciculus, and cortical‐spinal tract (Zarei et al., 2011). Discrepancies of effects across these studies may be explained by heterogeneities in sample size, age, clinical characteristics, medication status, processing protocols, and analytic method. A meta‐analysis may help identify the most prominent and replicable FA alterations of WM associated with OCD in TBSS studies.

Previous meta‐analyses of WM microstructure alterations in OCD included VBA studies (Eng et al., 2015; Peng et al., 2012; Radua, Grau, et al., 2014) or mixed VBA and TBSS studies (Piras et al., 2013) that had potential biases as noted above. The Enhancing Neuro‐Imaging Genetics through Meta‐Analysis (ENIGMA) consortium OCD working group performed a meta‐analysis of WM alterations in 874 patients with OCD compared to 789 healthy controls by analyzing TBSS data from 19 consortium sites rather than from all existing publications. They analyzed data from 25 WM ROIs rather than every WM voxel as reported in bioRxiv (Piras et al., 2019). Hu et.al integrated published TBSS studies of OCD that used either parametric and nonparametric tests and reported a meta‐analysis result derived from combined pediatric and adult subjects (Hu et al., 2020). Combining groups with significant differences in biological features such as over the course of adolescent maturation can lead to biases in findings.

Seed‐based *d* Mapping (SDM) software, a coordinate‐based meta‐analytic tool (www.sdmproject.com), can integrate various findings of previous neuroimaging studies to identify consistent conclusions through recreating the effect size maps according to reported peak coordinates with anisotropic kernels (Radua, Rubia, et al., 2014). It has advantages over the activation likelihood estimate or multilevel kernel density analysis methods for combining both positive and negative differences in the same map to avoid significantly opposite directions in the same voxel erroneously (Radua & Mataix‐Cols, 2009).

Therefore, in the present study, we conducted a quantitative whole‐brain meta‐analysis in SDM of peer‐reviewed and published TBSS studies of OCD, enabling results from individual studies to be weighted and controlled for multiple characteristics including clinical and imaging information, in order to identify replicable significant FA abnormalities. When the numbers of the studies were sufficient, we carried out subgroup meta‐analyses to explore WM alterations in adults, children and adolescents, studies with 3.0 T versus 1.5 T MRI scanners, and studies with statistical threshold correction for multiple comparisons. We also performed a multiple meta‐regression analysis to explore potential effects of age, sex, illness duration, medication status, age at onset, and clinical symptom severity on FA differences in patients with OCD.

## 2 MATERIAL AND METHODS

### 2.1 Study selection for the meta‐analysis

A systematic and extensive retrieval strategy was used to search for relevant literature published and “in press” articles in PubMed, EMBASE, Web of Science, and Science Direct up to 28 October 2020. In addition, manual searches were conducted among the reference sections of retrieved studies and review articles. Keywords were “tract‐based spatial statistical” or “TBSS” or “diffusion tensor imaging” or “diffusion magnetic resonance imaging” or “DTI” or “fractional anisotropy” or “FA”; AND “obsessive‐compulsive disorder” or “OCD.” The guidelines of Preferred Reporting Items for Systematic Reviews and Meta‐Analysis (Knobloch et al., 2011) were followed in the present study (Table S1).

Studies were included according to the following criteria: (a) an original, peer‐reviewed journal article; (b) used a sample with a primary diagnosis of OCD and HCs; (c) compared whole‐brain WM microstructural alterations between patients with OCD and HCs; and (d) applied the TBSS method to analyze whole‐brain voxel‐based comparisons of DTI data. We included studies regardless of whether they corrected for multiple comparisons, but required that FA alterations were reported in Talairach or Montreal Neurological Institute (MNI) brain coordinates. The corresponding authors of articles that did not report coordinates but were suitable for the current meta‐analysis were contacted by e‐mail asking for the pivotal information. Studies were excluded in the present meta‐analysis according to the following criteria: (a) case reports or reviews; (b) based on ROI analysis or small volume correction; (c) did not perform statistical comparisons between patients and controls; (d) did not perform statistical comparisons in FMRIB Software Library (FSL) (RRID:SCR_002823) with default non‐parametric permutation test (Nichols & Holmes, 2002); and (e) peak coordinates for relevant contrasts were not reported in manuscripts and were not provided after contacting the authors.

### 2.2 Data extraction and quality assessment

For all included studies, sample characteristics (sample size, age, and gender), clinical data (age at onset, illness duration, medication status, and symptom severity), scanning parameters, diffusivity measures obtained, and findings of localized FA alterations were extracted. We also extracted the coordinates of results and statistical values related to effect size, both of which were required for the following meta‐analysis.

The quality of included studies in our meta‐analysis was evaluated using a 15‐point checklist that was based on both the available clinical and demographic characteristics of the participants and the imaging methodology (Chen et al., 2016). Each criterion was scored as 1, 0.5, or 0 if the criteria were fully met, partially met, or unfulfilled, respectively. An assessed study that had a score above 7.5 points (50% of the total score) was included in the present meta‐analysis. The quality scores for each study are shown in Table S2. The study selection, data extraction, and quality assessment were independently performed by two authors (Q.L. and Y.J.Z.) and any inconsistent results were discussed and resolved by consensus.

### 2.3 SDM meta‐analysis

We conducted a voxel‐wise meta‐analysis in SDM software (version 5.15) (RRID:SCR_002554) according to a standard process. SDM used effect sizes to combine the reported peak coordinates of each included study and recreated the original maps of the effect size of group differences in FA by means of an anisotropic un‐normalized Gaussian kernel. Studies reporting no group differences were also included, which were estimated conservatively to have a null effect size. The modality of “DTI‐fractional anisotropy (TBSS)”, correlation template of “fractional anisotropy,” and mask of “TBSS” were chosen in SDM, which account for the anisotropy in the spatial covariance of the brain to increase the accuracy of the effect size maps. We used the default 20 mm full‐width at half‐maximum un‐normalized Gaussian kernel to assign indicators of proximity to reported coordinates (Radua, Rubia, et al., 2014). Following this, a mean map in MNI coordinates was created by a random‐effects meta‐analytic method, considering sample size, intra‐study variance, and between‐study heterogeneities. We used the default thresholds (voxel threshold *p* < .005 with peak *Z* > 1 and a cluster extent of 50 voxels) in SDM and reported the results in MNI coordinates. Subgroup meta‐analyses were also conducted when the number of studies was sufficient as described below.

A meta‐regression analysis was performed with age, sex ratio, age at onset, illness duration, percentage of patients receiving medication, and symptom severity considered as independent variables. To reduce the risk of spurious findings in the meta‐regression analyses, the significance threshold was defined as *p* < .0005 (Radua et al., 2012). The results from the regression analysis were only considered significant when those could be detected both in the significant overall slope and at higher or lower extremes of the regressor and were in regions where significant group differences were observed in the main meta‐analysis (Radua & Mataix‐Cols, 2009).

A whole‐brain jackknife sensitivity analysis for the meta‐analysis was conducted to test the replicability of results using the same threshold as the meta‐analysis. We iteratively repeated the main analysis *n* times (*n* = the number of total datasets), discarding a dataset each time, to determine whether the results of the meta‐analysis remained significant. The between‐studies heterogeneity of individual clusters from the results of the meta‐analysis was examined using a random‐effects model with *Q* statistics (*p* = .005, peak *Z* = 1, cluster extent = 50 voxels). Lastly, for each significant peak, the possibility of publication bias was examined using Egger's test to assess the asymmetry of funnel plots.

## 3 RESULTS

### 3.1 Review of 20 TBSS studies included in the study

**FIGURE 1:**
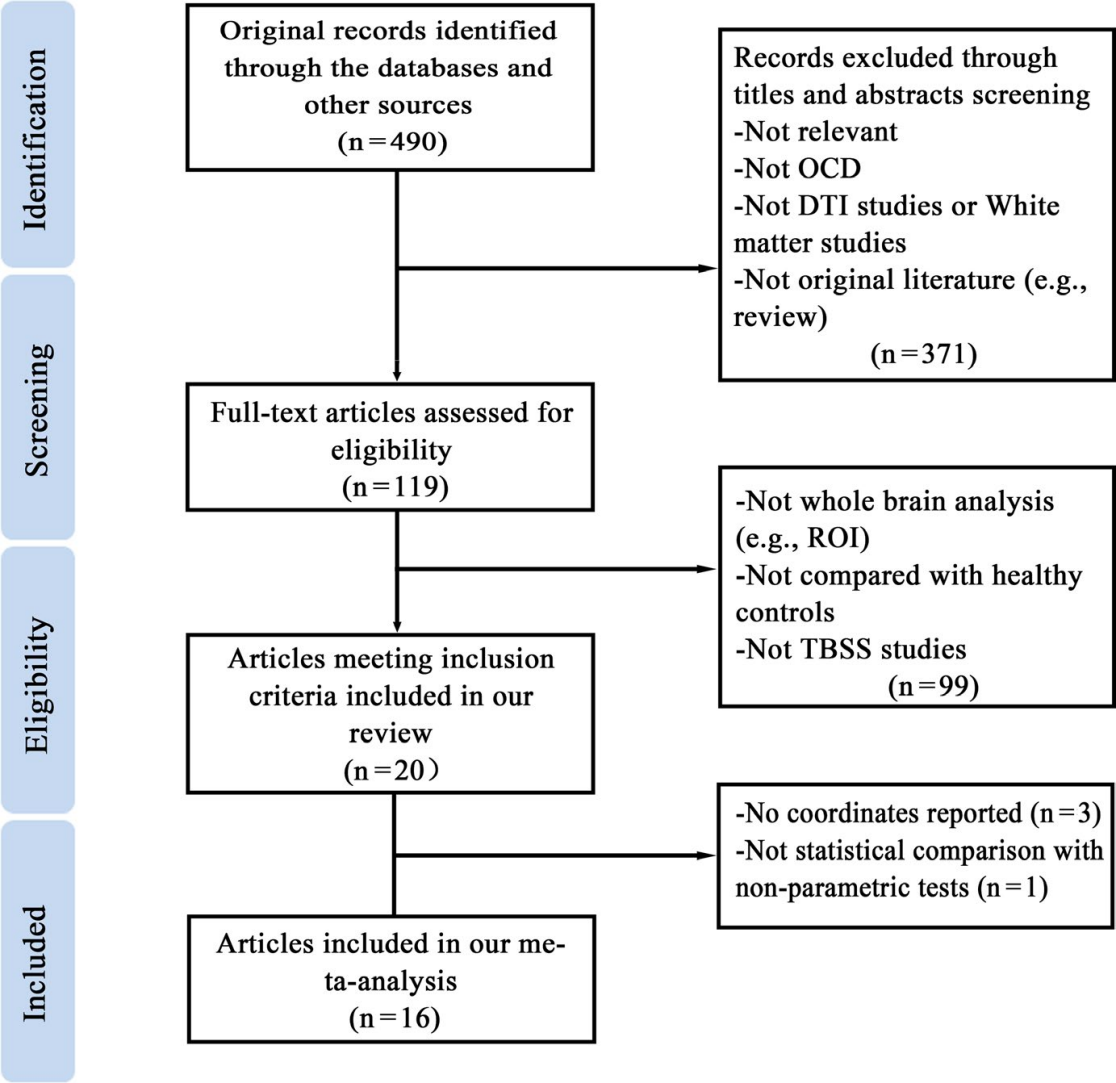
Flow diagram for the identification and exclusion of diffusion tensor imaging (DTI) studies enrolling patients with obsessive‐compulsive disorder (OCD) by using tract‐based spatial statistics (TBSS) analysis in FMRIB Software Library (FSL)

The flow chart of the identification and attrition of studies is provided in Figure 1. There were 20 TBSS studies included in the review (six studies recruiting child/adolescent patients (Ameis et al., 2016; Fitzgerald et al., 2014; Jayarajan et al., 2012; Rosso et al., 2014; Silk et al., 2013; Zarei et al., 2011) and 14 studies with adult patients (Benedetti et al., 2013; Bollettini et al., 2018; Bora et al., 2011; Fan, van den Heuvel, et al., 2015; Fontenelle et al., 2011; Gan et al., 2017; Hartmann et al., 2016; Hawco et al., 2017; Magioncalda et al., 2016; Nakamae et al., 2011; Salles Andrade et al., 2019; Spalletta et al., 2014; Yagi et al., 2017; Zhou et al., 2018)). Demographic information and clinical characteristics of participants in all 20 TBSS studies, as well as scanning parameters and main findings of FA analyses, are shown in Table 1 and Table 2. A detailed review of the 20 TBSS studies is provided in Appendix S1.

**TABLE 1:** Summary of demographic and clinical characteristics of 20 TBSS studies

Study	Patients with OCD							Healthy controls		
	No.	Female, *n* (%)	Age, y	YBOCS^a^	Duration, y	Onset, y	Drug status, *n* (%)^e^	No.	Female, *n* (%)	Age, y
Studies with adult patients with OCD included in the present meta‐analysis (*n* = 10)										
Salles Andrade et al. (2019)	23	8(34.8%)	39.7	27.7	25.2	14.5^c^	23(100%)	21	11(52.4%)	37.3
Zhou et al. (2018)^b^	52	24(46.2%)	32.2	25.3	3.8	28.4^c^	25(48.1%)	46	22(47.8%)	30.5
Bollettini et al. (2018)^b^	58	20(34.5%)	34.7	30.9	19.2	14.7	35(60.3%)	58	20(34.5%)	34.3
Yagi et al. (2017)	20	10(50%)	34.1	26.2	11.5	22.7	15(75.0%)	30	16(53.3%)	30.6
Hawco et al. (2017)	38	21(55.3%)	34.6	20.9	14.8	17.8	38(100%)	45	22(48.9%)	33.2
Gan et al. (2017)	24	9 (37.5%)	22.6	32.5	NA	NA	13(54.2%)	23	11(47.8%)	23.2
Magioncalda et al. (2016)	16	11(68.8%)	27.6	19.7	15.1	12.5^c^	13(81.3%)	18	10(55.6%)	28.2
Fan, van den Heuvel, et al. (2015)	44	22(50%)	38.5	21.5	NA	NA	0(0.0%)^d^	37	19(51.4%)	39.5
Benedetti et al. (2013)^b^	40	14(35%)	33.9	31.2	19.1	14.1	22(55.0%)	41	21(51.2%)	33.8
Nakamae et al. (2011)	30	16(53.3%)	31.6	23.8	6.7	25	0(0.0%)^d^	30	15(50%)	30.8
Studies with adult patients with OCD for which coordinates were not available (*n* = 3)										
Hartmann et al. (2016)	30	23(76.7%)	27	26.5	16	11^c^	17(56.7%)	30	21(70%)	28
Fontenelle et al. (2011)	9	2(22.2%)	26.2	28.5	14.7^f^	11.5	7(77.8%)	9	2(22.2%)	28
Bora et al. (2011)	21	10(47.6%)	34.4	19.2	NA	NA	10(47.6%)	29	15(51.7%)	31.4
Studies with adult patients with OCD that did not perform the statistical comparisons in FSL with non‐parametric permutation test (*n* = 1)										
Spalletta et al. (2014)	20	8(40%)	33.1	24.8	13.5	19.6^c^	12(60.0%)	20	8(40%)	35.2
Studies with child/adolescent patients with OCD included in the present meta‐analysis (*n* = 6)										
Ameis et al. (2016)	36	14(38.9%)	12.6	NA	NA	NA	13(36.1%)	62	25(40.3%)	10.8
Rosso et al. (2014)	17	6(35%)	14.1	17.1	5.2	8.8	14(82.4%)	19	6(32%)	13.6
Fitzgerald et al. (2014)	36	20(55.6%)	14.1	16.7	6.8^f^	7.3	18(50%)	27	16(59.3%)	14.7
Silk et al. (2013)	16	10(62.5%)	12.8	NA	NA	NA	14(87.5%)	22	6(27.3%)	11.2
Jayarajan et al. (2012)	15	7(46.7%)	14.1	21.5	1.4	12.7	13(86.7%)	15	7(46.7%)	14.3
Zarei et al. (2011)	26	12(46.2%)	16.6	19.5	5.3	11.2	16(61.5%)	26	12(46.2%)	16.

Abbreviations: FSL, FMRIB Software Library ; NA, not available; No., number; OCD, obsessive‐compulsive disorder; TBSS, tract‐based spatial statistics; YBOCS, Yale‐Brown Obsessive‐Compulsive Scale.

^a^ For the studies with children/adolescents with OCD, the Children's Yale‐Brown Obsessive‐Compulsive Scale (CYBOCS) was used to evaluate patients’ clinical symptoms.

^b^ These studies also conducted subgroup analyses based on medication status, but we just selected the results of the pooled analysis as the dataset in the present meta‐analysis.

^c^ Although these studies did not provide the exact age at onset, we calculated it by age minus duration of illness (age at onset = age − duration of illness).

^d^ The patients recruited in these studies were untreated for at least 4 weeks.

^e^ The percentage of patients received drug therapy.

^f^ Although these studies did not provide the exact duration of illness, we calculated it by age minus age at onset (duration of illness = age − age at onset).

**TABLE 2:** Summary of the scanning parameters and FA alterations of 20 TBSS studies

Study	Scanner (Tesla, T)	Slice thickness (mm)	Voxel size (mm^3^)	Directions	*p* value	Diffusivity measurements	Main findings of FA
Studies with adult patients with OCD included in the present meta‐analysis (*n* = 10)							
Salles Andrade et al. (2019)	3.0	2.5	NA	32	<.05, FWE	FA, MD	No significant FA alteration
Zhou et al. (2018)	3.0	3	2.0 × 2.1 × 3.0	33	<.05, TFCE	FA, MD	Decreased FA in CC (genu and body)
Bollettini et al. (2018)	3.0	2.3	NA	35	<.05, TFCE	FA, RD	Decreased FA in CC (genu, body and splenium), B.ATR, B.PTR, B. ALIC, B. RLIC, B.EC, B. UF, B.ILF, B. IFOF, B.SLF, B. parietal, CB, R.FCB, fornix (column and body), forceps minor, forceps major, B.MCP
Yagi et al. (2017)	3.0	NA	NA	30	<.05, TFCE	FA	No significant FA alteration
Hawco et al. (2017)	1.5	2.6	NA	23	<.05, TFCE	FA	Decreased FA in CC (genu)
Gan et al. (2017)	3.0	1.0	1.0 × 1.0 × 1.0	64	<.05, TFCE	FA, MD, AD, RD	Decreased FA in L.SLF, CC (splenium, body and genu) including L.ACR, L.SCR
Magioncalda et al. (2016)	3.0	NA	NA	42	<.05, TFCE	FA, MD, RD	No significant FA alteration
Fan, van den Heuvel, et al. (2015)	3.0	2.4	NA	30	<.05, TFCE	FA	No significant FA alteration
Benedetti et al. (2013)	3.0	2.3	NA	35	<.05, TFCE	FA, MD, AD, RD	Decreased FA in B.ACR, B. IFOF, R. ALIC, B.ATR, B. UF, B. forceps minor, R. CB, R.SLF, R.PTR, R.ILF, CC (body), and L.SCR
Nakamae et al. (2011)	1.5	3.0	NA	15	<.05, cluster‐forming threshold	FA	Decreased FA in CC (body)
Studies with adult patients with OCD for which coordinates were not available (*n* = 3)							
Hartmann et al. (2016)	3.0	3.5	NA	26	<.05, FWE	FA	Increased FA in cerebellum; decreased FA in CC (genu) and forceps minor
Fontenelle et al. (2011)	1.5	5	NA	6	<.05, corrected^a^	FA, MD	Decreased FA in SLF, IC (genu and posterior limb), and cerebral peduncle
Bora et al. (2011)	3.0	4.2	1.875 × 1.875 × 2.000	28	<.05, cluster‐forming threshold	FA, AD, RD	Decreased FA in CC (body)
Studies with adult patients with OCD that did not perform the statistical comparisons in FSL with non‐parametric permutation test (*n* = 1)							
Spalletta et al. (2014)	3.0	NA	1.8 × 1.8 × 1.8	30	<.001. uncorrected	FA	Decreased FA in CC (body) and L.SLF
Studies with child/adolescent patients with OCD included in the present meta‐analysis (*n* = 6)							
Ameis et al. (2016)	3.0	2.0	NA	60	<.05, FWE	FA, MD, AD, RD	No significant FA alteration
Rosso et al. (2014)	3.0	2.0	2.0 × 2.0 × 2.0	60	<.05, TFCE	FA, MD, AD, RD	Decreased FA in B. frontal lobe (ATR, UF, IFOF, forceps minor, and anterior corona radiata), B.CC (genu, body, splenium), R. cingulate, R. basal ganglia, R. posterior cerebral cortex, R. inferior frontal lobe, R. thalamus, R. caudate, and R. anterior IC (ATR)
Fitzgerald et al. (2014)	3.0	3.0	NA	15	<.05, TFCE	FA	No significant FA alteration
Silk et al. (2013)	3.0	3.0	NA	60	<.05, TFCE	FA, MD, AD, RD	No significant FA alteration
Jayarajan et al. (2012)	3.0	2.5	NA	32	<.05, TFCE	FA, AD, RD	No significant FA alteration
Zarei et al. (2011)	1.5	1.0	2.5 × 2.5 × 2.5	60	<.05, FDR	FA	Increased FA in L.ILF, B.SLF, R. IFOF, B.CST, CC (genu and splenium), B. forceps major, B. forceps minor, L. cingulum, and R. UF

Abbreviations: ACR, anterior corona radiata; AD, axial diffusivity; ALIC, anterior limb of internal capsule; ATR, anterior thalamic radiations; B, bilateral; CB, cingulum bundle; CC, corpus callosum; CR, corona radiata; CST, corticospinal tract; EC, external capsule; FA, fractional anisotropy; FCB, frontal portion of cingulum bundle; FDR, false discovery rate; FWE, family‐wise error rate; IC, internal capsule; IFOF, inferior frontal‐occipital fasciculus; ILF, inferior longitudinal fasciculus; MCP, middle cerebellar peduncula; MD, mean diffusivity; NA, not available; PTR, posterior thalamic radiations; RD, radial diffusivity; RLIC, retrolenticular part of internal capsule; SCR, superior corona radiata; SLF, superior longitudinal fasciculus; TFCE, threshold‐free cluster enhancement; UF, uncinate fasciculus.

^a^ The study did not report the specific correction method for the multiple comparisons.

### 3.2 Characteristics of 16 TBSS studies included in the meta‐analysis

In the 20 studies, peak coordinates of FA alteration could not be retrieved after contacting the authors in three studies (Bora et al., 2011; Fontenelle et al., 2011; Hartmann et al., 2016), and one study conducted statistical comparisons with parametric *t* tests in Statistical Parametric Mapping software (Spalletta et al., 2014). Therefore, 16 studies met criteria to be included in the present meta‐analysis (six studies recruiting child/adolescent patients (Ameis et al., 2016; Fitzgerald et al., 2014; Jayarajan et al., 2012; Rosso et al., 2014; Silk et al., 2013; Zarei et al., 2011) and 10 studies with adult patients (Benedetti et al., 2013; Bollettini et al., 2018; Fan, van den Heuvel, et al., 2015; Gan et al., 2017; Hawco et al., 2017; Magioncalda et al., 2016; Nakamae et al., 2011; Salles Andrade et al., 2019; Yagi et al., 2017; Zhou et al., 2018). No study used overlapping patient samples.

All the 16 studies included in the meta‐analysis explored diffusivity measurements of FA. Nine studies reported the mean diffusivity (MD) (Ameis et al., 2016; Fontenelle et al., 2011; Gan et al., 2017; Magioncalda et al., 2016; Nakamae et al., 2011; Rosso et al., 2014; Salles Andrade et al., 2019; Silk et al., 2013; Zhou et al., 2018), nine studies reported radial diffusivity (RD) (Ameis et al., 2016; Benedetti et al., 2013; Bollettini et al., 2018; Bora et al., 2011; Gan et al., 2017; Jayarajan et al., 2012; Magioncalda et al., 2016; Rosso et al., 2014; Silk et al., 2013), and seven studies reported axial diffusivity (AD) (Ameis et al., 2016; Benedetti et al., 2013; Bora et al., 2011; Gan et al., 2017; Jayarajan et al., 2012; Rosso et al., 2014; Silk et al., 2013) (Table 2 and Table S3). Thus, we performed our primary meta‐analysis on FA measurements available in all 16 studies. Across the 16 studies, there was no significant difference between patients with OCD (male, 267; female, 224) and HCs (male, 281; female, 239) in gender ratios (*p* = .91). A total of 491 patients with OCD (mean age: 27.8 years) and 520 HCs (mean age: 26.2 years), along with 53 coordinates of regions with significant case‐control differences in FA extracted from the 16 TBSS studies, were included in the present pooled meta‐analysis.

Among the 16 OCD studies in the meta‐analysis, two studies recruited all medicated patients (Hawco et al., 2017; Salles Andrade et al., 2019), two studies included patients unmedicated for at least 4 weeks before the MRI scanning (Fan, van den Heuvel, et al., 2015; Nakamae et al., 2011), and the remaining 12 studies enrolled patients with variable treatment status (Ameis et al., 2016; Benedetti et al., 2013; Bollettini et al., 2018; Fitzgerald et al., 2014; Gan et al., 2017; Jayarajan et al., 2012; Magioncalda et al., 2016; Rosso et al., 2014; Silk et al., 2013; Yagi et al., 2017; Zarei et al., 2011; Zhou et al., 2018). DTI was acquired using a 3.0 T MR scanner in 13 studies, while three studies used a 1.5 T MR scanner (Hawco et al., 2017; Nakamae et al., 2011; Zarei et al., 2011). All 16 studies in the meta‐analysis performed TBSS analysis with correction for multiple comparisons. We performed subgroup meta‐analyses in 10 studies with adult patients and 13 studies using a 3.0 T MR scanner.

### 3.3 Results of the pooled meta‐analysis

In the pooled meta‐analysis that included all 16 TBSS studies, patients with OCD showed no significant difference with regard to FA values compared to controls. Similar findings were observed in 15/16 datasets in jackknife sensitivity analyses (Table S4).

### 3.4 Subgroup meta‐analyses

**FIGURE 2:**
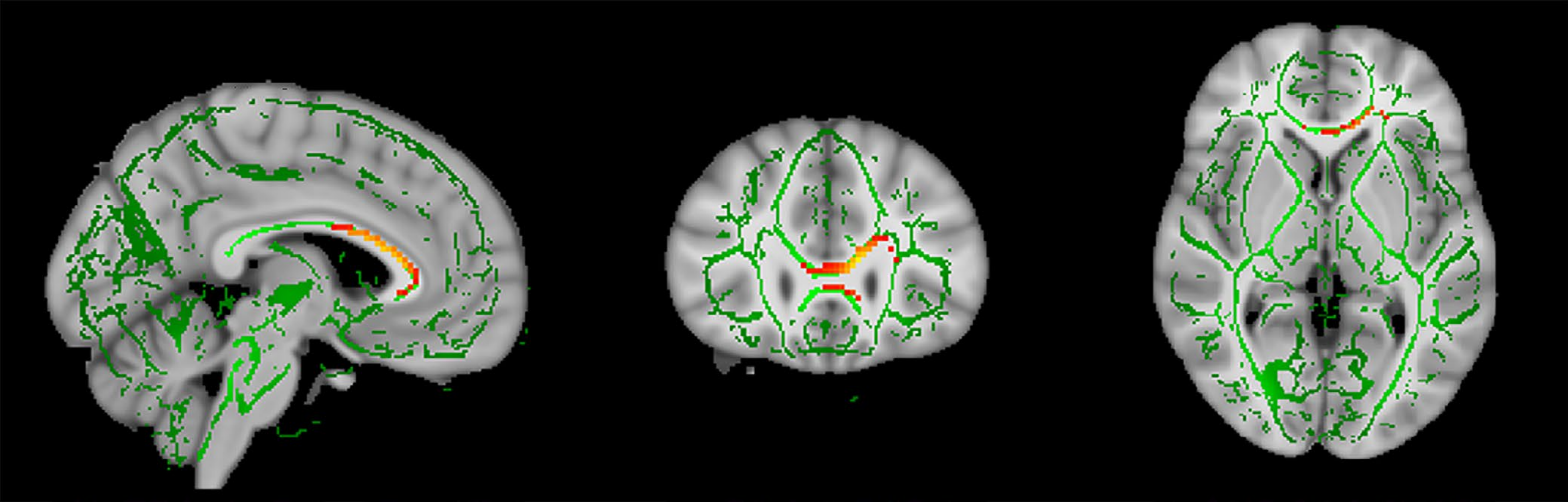
Decreased fractional anisotropy in the genu and anterior body of corpus callosum (CC) (warm color) was observed in the subgroup meta‐analysis in adult patients with obsessive‐compulsive disorder compared to controls

The OCD adult subgroup analysis included 10 studies that compared 345 patients with OCD (mean age: 33.6 years) with 349 controls (mean age: 32.7 years) that provided 12 extracted coordinates of regions with group differences in FA (Benedetti et al., 2013; Bollettini et al., 2018; Fan, van den Heuvel, et al., 2015; Gan et al., 2017; Hawco et al., 2017; Magioncalda et al., 2016; Nakamae et al., 2011; Salles Andrade et al., 2019; Yagi et al., 2017; Zhou et al., 2018). Sex ratios (adult patients with OCD: male, 190; female, 155. HCs: male, 182; female, 167) did not differ between groups (*p* = .44). Adult patients with OCD showed decreased FA in a region including the genu and anterior body of CC (*x* = −4, *y* = 28, *z* = 2; *p* = .00029, *Z* = −0.841; 480 voxels) compared to HCs (Table 3 and Figure 2). These findings were robust in the jackknife sensitivity analysis (Table S5). Analysis of heterogeneity revealed no significant between‐study heterogeneity. Egger's test of funnel plot asymmetry did not identify evidence of publication bias in the observed effects (Figure S1).

**TABLE 3:** Decreased fractional anisotropy in adult patients with obsessive‐compulsive disorder than health controls revealed by meta‐analyses of tract‐based spatial statistics studies

WM tract	MNI coordinates			SDM *Z* score	*p* value uncorrected	voxels *n*	Cluster breakdown (voxels, *n*)
	*x*	*y*	*z*				
Genu and anterior body of corpus callosum	−4	28	2	−0.841	.00029	480	Corpus callosum (434)
							Left median network, cingulum (16)
							Left anterior thalamic projections (15)
							Left striatum (9)
							Left inferior network, inferior frontal‐occipital fasciculus (6)

Abbreviations: MNI, Montreal Neurological Institute; SDM , Seed‐based *d* Mapping, WM, white matter.

A subgroup meta‐analysis of children and adolescents with OCD (six studies) was precluded given that the number of studies was too small to draw reliable conclusions. However, we reviewed the results of the six studies detailed in Appendix S1 and discussed the potential trajectory of FA alterations in pediatric patients with OCD. Subgroup meta‐analyses in 13 studies with 3.0 Tesla MR scanner, which included pediatric and adult patients with OCD, showed no differences in FA between patients with OCD and controls, consistent with the result of the pooled meta‐analysis with all 16 TBSS studies.

### 3.5 Meta‐regression analysis

**FIGURE 3:**
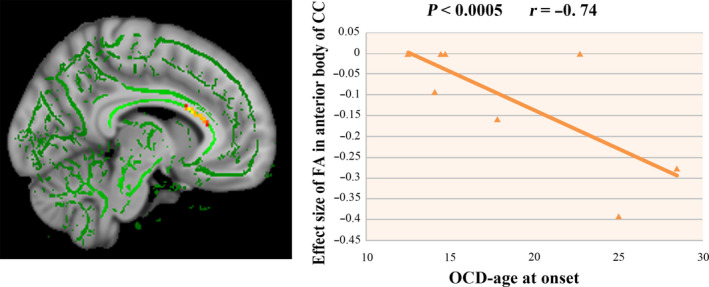
Fractional anisotropy (FA) value in the anterior body of corpus callosum (CC) was negatively related to age at onset in adult patients with obsessive‐compulsive disorder as revealed in the meta‐regression analysis

For the 10 studies in the subgroup meta‐analysis that recruited adult patients with OCD, age at onset was negatively correlated with FA in the anterior body of CC (*x* = −8, *y* = 22, *z* = 18; *r* = −0.74, *p* < .0005, *Z* = −1.294; 68 voxels) (Figure 3). We did not observe significant correlations between FA value and percentage of female patients with OCD, age, medication status, Yale‐Brown Obsessive‐Compulsive Scale (YBOCS), or illness duration in adult patients with OCD.

## 4 DISCUSSION

The key findings of the current meta‐analysis were the following: (a) no significant FA differences were observed in the pooled meta‐analysis of 16 studies which included both pediatric and adult patients; (b) FA was decreased in the region of the genu and anterior body of CC in adult patients with OCD; and (c) FA reductions in the anterior body of CC were associated with a later age of onset in adult patients with OCD. The current findings suggest that microstructural abnormalities in the anterior and central subdivisions of CC play an important role in the pathophysiology of adult patients with OCD.

The observation of altered WM fiber tracts in adults but not pediatric patients with OCD suggests different pathophysiological processes in pediatric cases including potential neurodevelopmental features. Clinical differences in pediatric OCD have been reported in clinical phenotype, gender (males are predominant in pediatric studies), and patterns of psychiatric comorbidity (Geller, 2006). Previous studies reported that child/adolescent OCD might be a developmental subtype of OCD (Geller et al., 1998; Huyser et al., 2009), consistent with our observation of greater inconsistencies of FA alterations and suggestions of atypical brain maturational trajectories evident in pediatric studies (Fitzgerald et al., 2014). Variable dysmaturation and inconsistent age‐related effects in pediatric patients may be a factor in the failure to find case‐control differences in the pooled sample (that included both adult and pediatric cases) when significant alterations were evident in adult patient studies.

In addition, the effects of head motion also should be taken into account. All included studies in our current meta‐analysis used the FMRIB diffusion Toolbox from the FSL processing software package (http://www.fmrib.ox.ac.uk/fsl) (RRID:SCR_002823) to analyze the DTI data. The motion and eddy current distortion correction were conducted by registering diffusion‐weighted images to a nondiffusion‐weighted image (*b* = 0) using affine registration before running TBSS, but this may not remove all effects of movements (Smith et al., 2006). Benedetti et al. (2016) and Bollettini et al. (2018) reported that manual inspection was also executed for head motion by trained researchers in their studies. Additionally, the pediatric study by Ameis et al. (2016) conducted thorough quality control to remove the effects of slice‐wise artifacts. Thus, movement artifacts as well as varying quality control procedures might add variance within samples‐especially the pediatric group and reduce sensitivity to case‐control differences in this age group.

Previous meta‐analyses in OCD including VBA studies (Eng et al., 2015; Peng et al., 2012; Radua, Grau, et al., 2014) or mixed VBA and TBSS studies (Piras et al., 2013) reported FA alterations in anterior midline tracts including CC though the direction varied. This variability might be due to alignment inaccuracies and less standard approaches for choosing a smoothing extent, which limits direct comparisons with the present results. Consistent with the report from the OCD working group of ENIGMA (Piras et al., 2019), we also identified decreased FA in CC in adult patients. However, we did not find FA alterations in posterior thalamic radiations or sagittal stratum as reported in the unpublished preprint from the ENIGMA study. In addition to differences in the patient cohort, different data processing protocols might contribute to these differences. The ENIGMA study performed a TBSS analysis using the ENIGMA‐DTI template and Johns Hopkins University ROIs atlas rather than using the default template in FSL which provides a whole‐brain voxel‐wise analysis. Compared with the study by Hu et al. (2020), we did not observe decreased FA in orbitofrontal WM, which again might be attributed to the different patient composition or study inclusion criteria. Our present study only included studies using the default permutation test in FSL to control the effects of different image analysis procedures across studies. In addition, Hu et al. (2020) treated a previous study (Benedetti et al., 2013) as two datasets where there were drug‐naive and drug‐treated patients compared to one group of controls, so the same control group was included twice in the initial statistical comparisons. In order to explore the potential relationship between the FA alteration and percentage of medicated patients with OCD, we treated this study as a single dataset rather than as two datasets with 0% and 100% medicated patients, respectively.

### 4.1 FA alterations in adult patients with OCD

The anterior segment of the CC has a relatively high FA perhaps increasing sensitivity to illness‐related changes. FA decrease in this region might be due to demyelination, reduced packing density, increased axon diameter, and other maturational and trophic factors (Le Bihan et al., 2001). Some studies reported decreased FA with increased RD but without alteration of AD in CC (Bollettini et al., 2018; Bora et al., 2011; Gan et al., 2017), suggesting greater diffusion alteration across tract membranes rather than along tracts, consistent with deficits of myelination in this region in OCD. From a genetic point of view, the neurotrophic tyrosine kinase receptor type 3 (NTRK3) gene has been associated with OCD symptoms, and NTRK3 gene is known to modulate the microstructural organization of CC (Alonso et al., 2008; Braskie et al., 2013). The oligodendrocyte lineage transcription factor 2 gene which is involved in myelination has also been associated with symptoms in patients with OCD (Vieira‐Fonseca et al., 2019). The myelin oligodendrocyte glycoprotein gene plays a vital role in the myelination process and has also been associated with OCD (Zai et al., 2004). The tumor necrosis factor‐α could induce WM injury by exerting cytotoxic effects on neurons and oligodendrocyte progenitors (Wang et al., 2014), and it has been shown to be increased in patients with OCD (Konuk et al., 2007) and negatively correlated with FA in CC (Benedetti et al., 2016).

The identified disruption of microstructural organization in CC might lead to a slower speed of interhemispheric communication (Cherbuin & Brinkman, 2006), and thus to cognitive deficits associated with the illness (Gonçalves et al., 2011). Consistent with this possibility, Garibotto et al. (2010) reported that FA alteration in CC was associated with cognitive dysfunction (decision‐making impairment and visuospatial deficits) in patients with OCD. Besides the microstructural alteration of FA, a previous study showed a shorter length of fiber bundles passing through the CC in OCD using fiber tractography methods, adding insight into anatomic alterations that might reduce the interhemispheric transfer of information in OCD (Gan et al., 2017). Further, callosal thickness and WM density analysis revealed that patients with OCD have thinner CC and lower CC density compared to controls (Di Paola et al., 2013), and thickness in the anterior part of CC has been associated with neuropsychological deficits and compulsive checking behaviors in OCD (Di Paola et al., 2013; Jaafari et al., 2013). Consistent with our findings, Radua, Grau, et al. (2014)) reported decreased FA in anterior midline tracts, which crossed between the body of CC and anterior part of cingulum bundle (CB) in their meta‐analysis with VBA studies in adult patients with OCD. Thus, the previously published literature, in aggregate, provides evidence for the importance of rostral CC alterations in the neurobiology of OCD.

The genu of the CC provides interhemispheric connections for the lateral and medial aspects of the prefrontal cortex, and the anterior body of CC connects homologous cerebral areas including premotor, supplementary motor, and motor regions. Thus, these CC regions provide the large bulk of interhemispheric neocortical connectivity of fronto‐striatal circuitry (Saito et al., 2008). Microstructural alterations in CC might underlie or result from previously identified structural and functional abnormalities in regions innervated by the rostral aspect of the CC in OCD. Patients with OCD have shown decreased GMV and WM volume in the bilateral prefrontal cortex (Heuvel et al., 2009; Togao et al., 2010). A recent mega‐analysis reported cortical thickness reductions in bilateral frontal cortex (Fouche et al., 2017) and Qin et al. (2019) found fewer fiber counts in the bilateral frontal cortex in OCD patients. Apart from structural abnormalities, functional studies also highlight the importance of trans‐callosal connectivity alterations in OCD. Adult patients with OCD have been reported to have decreased functional connectivity between the right ventral lateral prefrontal cortex and left insula (Chen et al., 2018) and decreased voxel‐mirrored homotopic connectivity in the precentral gyrus and orbitofrontal cortex (Deng et al., 2019). Additionally, increased functional frontal asymmetry in patients with OCD has been observed in electroencephalography studies (Perera et al., 2019).

Normally, FA in CC initially and rapidly increases during childhood, then reaches a plateau at approximately 11 years of age, and subsequently remains relatively stable and does not change or slowly decreases in adulthood (Keshavan et al., 2002; Lebel & Beaulieu, 2011; Lebel et al., 2008). In the present study, age at onset ranged from 12.5 to 28.4 years in studies with adult patients with OCD, and our meta‐regression analysis showed that lower FA in the anterior body of CC was associated with later age at onset in adult patients with OCD. This suggests a potentially important biological feature distinguishing pediatric and adult‐onset illness, potentially with later onset cases having illness‐related atrophic changes in rostral CC regions.

However, with this and other findings, the potential impact of psychotropic medications on WM needs to be taken into consideration when interpreting our findings. For example, patients with an earlier/pediatric age at onset may more likely receive drug therapy at an earlier age when refinement of brain circuitry and related cognitive processes are still active. Selective serotonin reuptake inhibitors, the most commonly prescribed medicine to treat OCD, possess neuroprotective and neurotrophic effects. These treatments also increase the serum level of brain‐derived neurotrophic factor (Hunsberger et al., 2009), which has been implicated in the progress of nervous system myelination (Xiao et al., 2010). Thus, treatments may impact WM microstructural organization in ways that require further investigation as has recently been shown with antipsychotic medications (Meng et al., 2019).

### 4.2 FA alterations in child and adolescent patients with OCD

In an important study, Fitgerald and colleagues observed two patterns of FA alteration in pediatric OCD: FA decrease in CC in patients 8–11 years of age, and FA increase in CC and CB in patients 16–19 years of age (Fitzgerald et al., 2014). This raises the possibility that decreased FA in child and early adolescent patients and increased FA in late adolescents with OCD that was observed by Fitzgerald et al. (2014) might reflect an altered neurodevelopmental trajectory of callosal fiber tracts. For example, delayed pruning of axonal connections into adolescence might account for this pattern of findings. Consistent with the possibility of abnormal maturation of CC fiber tracts in OCD, the mean age of pediatric patients with OCD in the study by Rosso et al. (2014) (14.1 years) was somewhat less than that of the study by Zarei et al. (2011) (16.6 years), and the former study reporting decreased FA and the latter reported increased FA in CC. In addition to these neurobiological considerations, complex age‐related effects in pediatric OCD may make it difficult to identify case‐control differences in pediatric patients.

Other studies have also reported neurodevelopmental abnormalities in pediatric patients with OCD. Rosenberg et al. (1997) reported that the age‐related increased length and size of CC and increased volume of anterior cingulate volumes in HCs were absent in pediatric patients with OCD (Rosenberg & Keshavan, 1998). Huyser et al. (2009) identified developmentally mediated abnormalities of CSTC and limbic circuitry in pediatric OCD by reviewing both structural and functional neuroimaging studies, proposing an alteration of myelinization and neurodevelopmental pruning of neural networks in OCD. These findings and the heterogeneity of pediatric OCD findings with regard to WM studies highlight the need for more intensive and longitudinal studies of neurodevelopment in pediatric OCD.

### 4.3 Limitations

A majority of studies in the current meta‐analysis (12/16) recruited patients receiving a variety of doses and types of medications. Thus, subgroup meta‐analysis with medicated and medication‐naive patients could not be performed to explore potential medication effects on FA in OCD. Some cross‐sectional and potentially underpowered TBSS studies found there were no significant FA differences in CC between medicated and unmedicated OCD groups (Bora et al., 2011; Gan et al., 2017; Zhou et al., 2018), while the study by Bollettini et al. (2018) reported that some drug treatments may have a robust effect on the observed FA decrease in CC. As illness may be associated with reduced FA and drug treatments may enhance myelination and FA or decrease them (Fan, Bhatt, et al., 2015; Konopaske et al., 2008), sorting out medication effects on the brain in greater detail is needed to understand illness‐associated brain alterations. In this context, drug dose and type may have increased variability of findings across studies, with a potential effect of reducing the ability to consistently see WM alterations in regions outside rostral CC. Future studies are needed to clarify drug effects on brain anatomy and its development in OCD patients.

Other limitations of our study also need to be acknowledged. First, there are heterogeneities of MRI scanning parameters across studies that may impact our findings, though we note that the same negative effect as our primary pooled analysis was seen in the subgroup analysis of studies using a 3.0 T MRI scanner. Second, additional diffusivity parameters (MD, AD, and RD) were reported in some studies, but too few to perform a meta‐analysis of these features that might provide a more comprehensive picture of the mechanisms underlying WM alteration in OCD. Third, the number of TBSS studies focusing on pediatric patients with OCD was too small to conduct a quantitative subgroup meta‐analysis of pediatric patients. Fourth, based on a tensor model and a limited number of diffusion directions, an analysis of DTI data has limited accuracy in regions of crossing tracts thus potentially creating false tracts (Alexander et al., 2007), which TBSS method could not resolve this problem yet (Smith et al., 2006). Recently developed diffusion models (e.g., multi‐compartment model) and protocols (e.g., neurite orientation dispersion and density imaging and high‐angular‐resolution diffusion imaging) may help address this issue in future research. DTI images acquired with echo‐planar readout have inevitable susceptibility‐induced geometric distortions. Although TOPUP, a tool in FSL, is available to provide robust and effective performance in correcting distortions, advanced DTI scanning protocols acquiring images in two opposite phase encoding directions (right‐to‐left and left‐to‐right) may further minimize the distortions. In addition, the TBSS method cannot fully remove all movement‐related effects (Smith et al., 2006).

## 5 CONCLUSION

In conclusion, our study revealed robust disruptions of WM in the CC (genu and anterior body) reflected in a significant FA decrease in adult OCD. Adult patients with older age at OCD onset had more severe FA alterations in the anterior body of CC. For pediatric OCD, alterations in neurodevelopmental maturation may contribute to inconsistent patterns of FA alteration relative to matched controls during adolescence.

## CONFLICT OF INTEREST

Dr. Sweeney has consulted to VeraSci. Other authors declare no biomedical financial interests or potential conflicts of interest.

## AUTHOR CONTRIBUTION

FL and QYG designed the study. QL and YJZ drafted the manuscript. QL, YJZ, ZXH, YG, JYL, LKL, and WFY conducted literature searches, data management, and analysis, as well as data interpretation. QL, YJZ, LKL, and WFY made the figures and tables. JAS, FL, and QYG critically revised the manuscript. All authors approved the final manuscript.

## Supporting information

Appendix S1

## Data Availability

Data sharing is not applicable to this article as no new data were created in the current study.
