# Supplementary material for: Microstructural white matter abnormalities in pediatric and adult obsessive‐compulsive disorder: A systematic review and meta‐analysis
Source: Brain Behav. 2020 Dec 3;11(2):e01975. doi: 10.1002/brb3.1975 (PMC7882176; doi:10.1002/brb3.1975)
Supplement: Supplementary file 1 — Appendix S1 [file BRB3-11-e01975-s001.doc]

**Appendix S1**

**Microstructural white matter abnormalities in pediatric and adult obsessive-compulsive disorder: a systematic review and meta-analysis**

Qian Li^a,b,c#^, Youjin Zhao^a,b,c#^, Zixuan Huang^a,d^, Yi Guo^a,b,c^, Jingyi Long^a^,^b,c^, Lekai Luo^a,b,c^, Wanfang You^a,b,c^, John A. Sweeney^a,e^, Fei Li^a,b,c*^, Qiyong Gong^a,b,c*^

**Author Affiliations:**

^a^ Huaxi MR Research Center (HMRRC), Department of Radiology, West China Hospital of Sichuan University, Chengdu 610041, Sichuan, P.R. China.

^b^ Research Unit of Psychoradiology, Chinese Academy of Medical Sciences, Chengdu 610041, Sichuan, P.R. China.

^c^ Functional and Molecular Imaging Key Laboratory of Sichuan University, Chengdu 610041, Sichuan, P.R. China

^d^ Medical Imaging Technology Department, West China School of Medicine, Sichuan University, Chengdu 610041, Sichuan, P.R. China.

^e^ Department of Psychiatry, University of Cincinnati, Cincinnati, OH, United States.

^#^ Qian Li and Youjin Zhao contributed to the work equally.

^*^ Corresponding authors: Fei Li, M.D., Ph.D. (charlie_lee@qq.com) and Qiyong Gong, M.D., Ph.D. (qiyonggong@hmrrc.org.cn), Huaxi MR Research Center (HMRRC), Department of Radiology, West China Hospital of Sichuan University, No. 37 Guo Xue Lane, Chengdu 610041, Sichuan, P.R. China.

**Review of 20 TBSS studies included in the study**

*1. TBSS studies in adult patients with OCD*

Fourteen out of all 20 tract-based spatial statistics (TBSS) studies in the review recruited adult patients with obsessive-compulsive disorder (OCD), most of which reported decreased fractional anisotropy (FA). The earliest TBSS study recruited 30 drug-free adult patients with OCD and reported decreased FA in the anterior body of corpus callosum (CC) without a statistically significant correlation with the Yale-Brown Obsessive-Compulsive Scale (YBOCS) score^1^. Then, Bora et al. also reported decreased FA in the body of CC in adult patients with OCD compared with healthy controls (HCs)^2^, as well as increased radial diffusivity (RD), but no differences in axial diffusivity (AD), indicating that the disrupted with matter (WM) microstructural organization might be driven by abnormal myelination. There were no significant differences between male and female patients and medicated and unmedicated patients^2^. They did not report an association between FA of the CC and depression or anxiety ratings.

Zhou et al. identified significantly decreased FA in the genu and body of CC and no statistically differences in mean diffusivity (MD) in comparison to controls, and identified no brain areas with significant WM differences between medicated and medication-naive patients or correlations between FA alterations and clinical symptoms (YBOCS scores)^3^.

Gan et al. combined TBSS analysis and fiber tractography investigating association and commissural fibers^4^. They reported decreased FA in CC, anterior corona radiata (ACR), superior corona radiata (SCR), superior longitudinal fascicule (SLF), and fiber bundles via the SLF and increased RD in CC in patients with OCD, as well as shorter lengths of fiber bundles in the ACR, SCR, and CC. Concordant with findings from the subgroup analysis by Bora et al.^2^ and Zhou et al.^3^, there were no significant differences between medicated and medication-naive patients^4^, though samples for the comparison were not large.

As opposed to the results of the previous 3 studies^2-4^, Benedentti et al.^5^ and Bollettini et al.^6^ reported that WM abnormalities in OCD might be attributed to the effect of antipsychotic medication. Benedentti et al. observed that there were decreased FA in ACR, forceps minor, inferior frontal-occipital fasciculus (IFOF), and uncinate fasciculus (UF) in medicated patients compared to the HCs and decreased FA in IFOF, SLF, and inferior longitudinal fasciculus (ILF) in medicated patients compared to the medication-naive patients, while there were no significant differences between the medication-naive patients and HCs. Bollettini and colleagues found a widespread FA reduction and RD increase mainly in the CC, anterior thalamic radiations (ATR), posterior thalamic radiations, and anterior limb of the internal capsule (ALIC)^6^ in the whole patients group than controls and the currently treated patients showed FA decrease in similar regions compared to drug-naive patients, previously treated patients, and HCs, respectively.

Hawco et al. investigated the effects of age on FA in adult patients with OCD and found there was an accelerated reduction in FA with age significantly shown in the SLF^7^. They identified FA reductions in the genu of the CC that was more marked in female than male patients.

Fontenelle et al. found reduced FA and increased MD in the genu and posterior limb of the internal capsule (PLIC), corona radiata, and SLF in OCD^8^. Another study conducted by Spalletta and colleagues reported decreased FA in the body of CC and SLF in patients without comorbid other psychiatric disorders, and that semantic fluency scores were positively correlated with FA in the right posterior corona (PCR) radiata and the left cortical-spinal tract (CST)^9^.

Hartmann et al. suggested that the cerebellum might also be involved in the pathophysiology of OCD^10^. FA reductions in the cerebellum and FA increase in forceps minor were shown in their study, which were not attributed to the WM hyperintensity and volume.

To date, 4 TBSS studies reported no between-group differences in FA between adult patients with OCD and controls^11-14^. Yagi et al. did not find the FA differences between patients and controls or correlations between FA and clinical severity in medicated patients^13^. Fan et al. compared unmedicated patients with HCs, and also did not observe group differences in FA or correlations between FA and clinical measures^11^. Magioncalda et al. also did not detect differences in FA between patients and controls, but did report increased MD in PCR, ACR, SLF, CC, and PLIC and increased RD in the latter 4 areas in OCD relative to controls^12^. Additionally, Magioncalda and colleagues reported that cognitive performance was negatively correlated with MD in PLIC in adult patients with OCD. The latest TBSS study in OCD was conducted by Andrade and colleagues^14^. They did not identify the between-group differences in FA at a whole-brain level. However, in their regions of interest (ROIs) analysis, they found FA reductions in the left cingulum bundle (CB) in patients with OCD relative to HCs. Additionally, they reported negative correlations between the YBOCS obsession sub-ratings and illness duration with FA value in CB.

*2. TBSS studies in child or adolescent patients with OCD*

Only 6 studies assessed FA alterations in child or adolescent patients with OCD using the TBSS method^15-20^. Zarei and colleagues included 26 pediatric patients with OCD (mean age 16.6 years) and 26 matched HCs^20^ and found higher FA in patients with OCD than controls in CC (splenium and genu), bilateral SLF, CST, and forceps major and minor, left ILF and cingulum, right IFOF and UF. Additionally, positive correlations between FA and clinical symptom ratings were detected in association and projection WM tracts (including UF, CST, and SLF).

Rosso and colleagues recruited 17 pediatric patients (mean age 14.1 years) with a relative earlier onset of illness (average 8.8 years), analyzed 4 diffusion parameters (FA, MD, RD, and AD), and reported decreased FA in CC, frontal cortex, posterior parietal and occipital lobes and extending to subcortical areas (thalamus and putamen)^18^. Compared to HCs, patients exhibited significantly higher RD in the right frontal cortex and right body of the CC, indicating potential demyelination. Additionally, they reported that earlier age at onset of OCD was associated with lower FA in the right thalamus and with higher RD in the right CC, suggesting that age at onset might be a moderator of WM abnormalities. As opposed to the significant correlation findings in Zarei’s study^20^, Rosso et al. did not detect significant correlations between FA, RD and symptom severity^18^. Noteworthy, Rosso et al. pointed out that decreased FA in the splenium of CC^18^ might be affected by medication treatment in their study.

Another study with a sample of 36 patients across a wide age range (8 to 19 years) was conducted by Fitzgerald and colleagues to characterize the development of WM tracts in child and adolescent patients with OCD^16^. When controlling for age, there were no significant between-group differences in FA based on both whole-brain and ROI analysis. However, in an analysis of group by age interaction effect, they found an age-related increase of FA in the ROIs, including anterior part of CC, CB, and ALIC in patients and a positive correlation between FA in anterior CB and symptom severity of OCD at ROIs level. Interestingly, in the subgroup analysis, Fitzgerald et al. reported decreased FA in the genu and anterior body of CC in patients with OCD aged 8 to 11 years, while increased FA in the anterior body of CC and anterior part of CB in patients aged 16 to 19 years, highlighting the complexity of developmental effects on the maturation of brain WM pathways in this disorder.

Another 3 studies showed that there were no statistical differences in FA between pediatric patients with OCD and matched controls. Jayarajan and colleagues reported that FA was not significantly different in pediatric OCD (mean age: 14.1 years) and controls^17^. Instead, patients with OCD showed higher AD in CC (genu, body, and splenium), bilateral SLF, left ILF, bilateral CB, ALIC, and ATR, left PLIC, and middle cerebellar peduncle. Increased RD in CC (genu), SLF, ILF, UF, ATR, IFOF, and cerebellar peduncles were also observed in OCD, which might be an indication of atypical fiber packing and myelination. Jayarajan et al. failed to find any correlation between diffusion parameters and clinical profiles (severity, medication dosage, and ill duration). Silk and colleagues reported increased AD in the genu and splenium of CC but no significant correlations with clinical ratings in child and adolescent patients with OCD (mean age 12.8 years)^19^. One recent study recruiting 36 patients with OCD (mean age 12.6 years) found no significant differences in FA and RD between patients and controls, while the AD in the left thalamus extending into the internal capsule was significantly lower in patients than controls^15^.

**Table S1. PRISMA 2009 Checklist*.**

| **Section/topic** | **#** | **Checklist item** | **Reported on page #** |
| --- | --- | --- | --- |
| **TITLE** | | |  |
| Title | 1 | Identify the report as a systematic review, meta-analysis, or both. | 1 |
| **ABSTRACT** | | |  |
| Structured summary | 2 | Provide a structured summary including, as applicable: background; objectives; data sources; study eligibility criteria, participants, and interventions; study appraisal and synthesis methods; results; limitations; conclusions and implications of key findings; systematic review registration number. | 3-4 |
| **INTRODUCTION** | | |  |
| Rationale | 3 | Describe the rationale for the review in the context of what is already known. | 5-7 |
| Objectives | 4 | Provide an explicit statement of questions being addressed with reference to participants, interventions, comparisons, outcomes, and study design (PICOS). | 7-8 |
| **METHODS** | | |  |
| Protocol and registration | 5 | Indicate if a review protocol exists, if and where it can be accessed (e.g., Web address), and, if available, provide registration information including registration number. | 9 |
| Eligibility criteria | 6 | Specify study characteristics (e.g., PICOS, length of follow-up) and report characteristics (e.g., years considered, language, publication status) used as criteria for eligibility, giving rationale. | 9-10 |
| Information sources | 7 | Describe all information sources (e.g., databases with dates of coverage, contact with study authors to identify additional studies) in the search and date last searched. | 9-10 |
| Search | 8 | Present full electronic search strategy for at least one database, including any limits used, such that it could be repeated. | 9-10 |
| Study selection | 9 | State the process for selecting studies (i.e., screening, eligibility, included in systematic review, and, if applicable, included in the meta-analysis). | 9-10 |
| Data collection process | 10 | Describe method of data extraction from reports (e.g., piloted forms, independently, in duplicate) and any processes for obtaining and confirming data from investigators. | 10 |
| Data items | 11 | List and define all variables for which data were sought (e.g., PICOS, funding sources) and any assumptions and simplifications made. | 10 |
| Risk of bias in individual studies | 12 | Describe methods used for assessing risk of bias of individual studies (including specification of whether this was done at the study or outcome level), and how this information is to be used in any data synthesis. | 11-12 |
| Summary measures | 13 | State the principal summary measures (e.g., risk ratio, difference in means). | 11 |
| Synthesis of results | 14 | Describe the methods of handling data and combining results of studies, if done, including measures of consistency (e.g., I^2^) for each meta-analysis. | 11 |
| Risk of bias across studies | 15 | Specify any assessment of risk of bias that may affect the cumulative evidence (e.g., publication bias, selective reporting within studies). | 11-12 |
| Additional analyses | 16 | Describe methods of additional analyses (e.g., sensitivity or subgroup analyses, meta-regression), if done, indicating which were pre-specified. | 12-13 |
| **RESULTS** | | |  |
| Study selection | 17 | Give numbers of studies screened, assessed for eligibility, and included in the review, with reasons for exclusions at each stage, ideally with a flow diagram. | 13 |
| Study characteristics | 18 | For each study, present characteristics for which data were extracted (e.g., study size, PICOS, follow-up period) and provide the citations. | 13-14 |
| Risk of bias within studies | 19 | Present data on risk of bias of each study and, if available, any outcome level assessment (see item 12). | 15-16 |
| Results of individual studies | 20 | For all outcomes considered (benefits or harms), present, for each study: (a) simple summary data for each intervention group (b) effect estimates and confidence intervals, ideally with a forest plot. | 15 |
| Synthesis of results | 21 | Present results of each meta-analysis done, including confidence intervals and measures of consistency. | 15 |
| Risk of bias across studies | 22 | Present results of any assessment of risk of bias across studies (see Item 15). | 15 |
| Additional analysis | 23 | Give results of additional analyses, if done (e.g., sensitivity or subgroup analyses, meta-regression [see Item 16]). | 15-16 |
| **DISCUSSION** | | |  |
| Summary of evidence | 24 | Summarize the main findings including the strength of evidence for each main outcome; consider their relevance to key groups (e.g., healthcare providers, users, and policy makers). | 17-23 |
| Limitations | 25 | Discuss limitations at study and outcome level (e.g., risk of bias), and at review-level (e.g., incomplete retrieval of identified research, reporting bias). | 23-25 |
| Conclusions | 26 | Provide a general interpretation of the results in the context of other evidence, and implications for future research. | 25 |
| **FUNDING** | | |  |
| Funding | 27 | Describe sources of funding for the systematic review and other support (e.g., supply of data); role of funders for the systematic review. | 2 |

*Note: Moher D, Liberati A, Tetzlaff J, Altman DG, The PRISMA Group (2009). Preferred Reporting Items for Systematic Reviews and Meta-Analyses: The PRISMA Statement. PLoS Med 6(7): e1000097. doi:10.1371/journal.pmed1000097. For more information, visit www.prisma-statement.org.

**Table S2. Imaging methodology quality assessment checklist for studies included in the pooled meta-analysis.**

| **15-point checklist** | Andrade | Zhou | Bollettini | Gan | Yagi | Hawco | Magioncalda | Ameis | Fan | Rosso | Fitzgerald | Silk | Benedetti | Jayarajan | Nakamae | Zarei |
| --- | --- | --- | --- | --- | --- | --- | --- | --- | --- | --- | --- | --- | --- | --- | --- | --- |
|  | 14 | 3 | 6 | 4 | 13 | 7 | 12 | 15 | 11 | 18 | 16 | 19 | 5 | 17 | 1 | 20 |
| **Category 1: Subjects** |  |  |  |  |  |  |  |  |  |  |  |  |  |  |  |  |
| 1. Patients were evaluated prospectively, specific diagnostic criteria were applied, and demographic data were reported | 1 | 1 | 1 | 1 | 1 | 1 | 1 | 1 | 1 | 1 | 1 | 1 | 1 | 1 | 1 | 1 |
| 2. Healthy comparison participants were evaluated prospectively; psychiatric and medical illnesses were excluded | 1 | 1 | 1 | 1 | 1 | 1 | 1 | 1 | 1 | 1 | 1 | 1 | 1 | 1 | 1 | 1 |
| 3. Important variables (e.g., age, gender, medication status, comorbidity, and subtype) were checked either via stratification or statistics | 1 | 0.5 | 0.5 | 1 | 0.5 | 0.5 | 0.5 | 1 | 0.5 | 0.5 | 0.5 | 0.5 | 0.5 | 0.5 | 0.5 | 1 |
| 4. All patients were comorbidity free | 0.5 | 1 | 1 | 1 | 0.5 | 0.5 | 1 | 0.5 | 1 | 0.5 | 0.5 | 0.5 | 1 | 0.5 | 1 | 0.5 |
| 5. All patients were medication naive or free | 0 | 0.5 | 0.5 | 0.5 | 0.5 | 0 | 0.5 | 0.5 | 1 | 0.5 | 0.5 | 0.5 | 0.5 | 0.5 | 1 | 0.5 |
| 6. Sample size per group: ≥ 20, scores 1; ≥ 10, scores 0.5 | 1 | 1 | 1 | 1 | 1 | 1 | 0.5 | 1 | 1 | 0.5 | 1 | 0.5 | 1 | 0.5 | 1 | 1 |
| **Category 2: Methods for image acquisition and analysis** |  |  |  |  |  |  |  |  |  |  |  |  |  |  |  |  |
| 7. Magnet strength: 3T, scores 1; 1.5T, scores 0.5 | 1 | 1 | 1 | 1 | 1 | 0.5 | 1 | 1 | 1 | 1 | 1 | 1 | 1 | 1 | 0.5 | 0.5 |
| 8. Number of diffusion gradient directions: ≥ 20, scores 1; ≥ 12, scores 0.5 | 1 | 1 | 1 | 1 | 1 | 1 | 1 | 1 | 1 | 1 | 0.5 | 1 | 1 | 1 | 0.5 | 1 |
| 9. MRI slice-thickness ≤3 mm | 1 | 1 | 1 | 1 | 0 | 1 | 0 | 1 | 1 | 1 | 1 | 1 | 1 | 1 | 1 | 1 |
| 10. Technical factors such as magnet strength, number of diffusion gradient direction, voxel resolution *etc.* are clearly declared | 1 | 0.5 | 0.5 | 1 | 0.5 | 0.5 | 0.5 | 0.5 | 0.5 | 1 | 1 | 0.5 | 0.5 | 0.5 | 0.5 | 1 |
| 11. The imaging technique used was clearly described so that it could be reproduced | 1 | 1 | 1 | 1 | 1 | 1 | 1 | 1 | 1 | 1 | 1 | 1 | 1 | 1 | 1 | 1 |
| 12. Whole brain analysis was automated without a previously defined region | 1 | 1 | 1 | 1 | 1 | 1 | 1 | 1 | 1 | 1 | 1 | 1 | 1 | 1 | 1 | 1 |
| 13. Coordinates of decreased or increased FA reported in a standard space | 1 | 1 | 1 | 1 | 1 | 1 | 1 | 1 | 1 | 1 | 1 | 1 | 1 | 1 | 1 | 1 |
| **Category 3: Results and conclusions** |  |  |  |  |  |  |  |  |  |  |  |  |  |  |  |  |
| 14. Statistical results were corrected for multiple comparison scores 1, uncorrected scores 0.5 | 1 | 1 | 1 | 1 | 1 | 1 | 1 | 1 | 1 | 1 | 1 | 1 | 1 | 1 | 1 | 1 |
| 15. Conclusions were consistent with the results obtained, and the limitations were discussed | 1 | 1 | 1 | 1 | 1 | 1 | 1 | 1 | 1 | 1 | 1 | 1 | 1 | 1 | 1 | 1 |
| **Total score** | 13.5 | 13.5 | 13.5 | 14.5 | 12 | 12 | 12 | 13.5 | 14 | 13 | 13 | 12.5 | 13.5 | 12.5 | 13 | 13.5 |

**Table S3. Main findings of other diffusivity measures (MD, AD, and RD)**

| **Study** | **Diffusivity measures** | **Main findings of MD** | **Main findings of AD** | **Main findings of RD** |
| --- | --- | --- | --- | --- |
| **Studies with adult patients with OCD included in the present meta-analysis (n = 10)** | | | | |
| Andrade.2019^14^ | FA, MD | No significant MD alteration | —— | —— |
| Zhou.2018^3^ | FA, MD | No significant MD alteration | —— | —— |
| Bollettini.2018^6^ | FA, RD | —— | —— | Increased RD in column and body of the fornix, B.ATR, B.PTR, B. ALIC, B. RLIC, B. EC, B. UF, B.ILF, B. IFOF, B. parietal portion of CB, R. frontal portion of CB, CC (genu and body), Forceps minor |
| Yagi.2017^13^ | FA | —— | —— | —— |
| Hawco.2017^7^ | FA | —— | —— | —— |
| Gan.2017^4^ | FA, MD, AD, RD | No significant MD alteration | No significant AD alteration | Increased RD in CC (genu and body) |
| Magioncalda.2016^12^ | FA, MD, RD | Increased MD in B.PCR, L.ACR, L. PLIC | —— | Increased RD in R.PCR, L.ACR |
| Fan.2015^11^ | FA | —— | —— | —— |
| Benedetti.2013^5^ | FA, MD, AD, RD | Increased MD in B. IFOF, L.ATR, L. UF, B.SLF, Forceps minor, L.EC, R.ILF | No significant AD alteration | Increased RD in L.SLF, L. CB, Forceps minor, B. IFOF, L.SCR, B.ATR |
| Nakamae.2011^1^ | FA | —— | —— | —— |
| **Studies with adult patients with OCD for which coordinates were not available (n = 3)** | | | | |
| Hartmanm.2016^10^ | FA | —— | —— | —— |
| Fontenelle.2011^8^ | FA, MD | Increased MD in cerebral peduncle, IC (genu and posterior limb), CR | —— | —— |
| Bora.2011^2^ | FA, AD, RD | —— | No significant AD alteration | Increased RD in CC (body) |
| **Studies with adult patients with OCD that did not perform the statistical comparisons in FSL with non-parametric permutation test (n = 1)** | | | | |
| Spalletta.2014^9^ | FA | —— | —— | —— |
| **Studies with child/adolescent patients with OCD included in the present meta-analysis (n = 6)** | | | | |
| Ameis.2016^15^ | FA, MD, AD, RD | No significant MD alteration | Decreased AD in L. thalamus, IC | No significant RD alteration |
| Rosso.2014^18^ | FA, MD, AD, RD | No significant MD alteration | No significant AD alteration | Increased RD in R. frontal cortex, CC (body) |
| Fitzgerald.2014^16^ | FA | —— | —— | —— |
| Silk.2013^19^ | FA, MD, AD, RD | No significant MD alteration | Decreased AD in CC (genu and splenium) | No significant RD alteration |
| Jayarajan.2012^17^ | FA, AD, RD | —— | Increased AD in CC (genu, body and splenium), B.SLF, L.ILF, B. CB, B.ATR, B. ALIC, L. PLIC, middle cerebellar peduncle | Increased RD in CC (genu), B.SLF, L.ILF, B. UF, B.ATR, B. IFOF, L. PLIC, superior and middle and inferior cerebellar peduncle |
| Zarei.2011^20^ | FA | —— | —— | —— |

Abbreviation: ACR, anterior corona radiata; AD, axial diffusivity; ALIC, anterior limb of internal capsule; ATR, anterior thalamic radiations; B, bilateral; CC, corpus callosum; CB, cingulum bundle; CR, corona radiata; EC, external capsule; FA, fractional anisotropy; IC, internal capsule; IFOF, inferior frontal-occipital fasciculus; ILF, inferior longitudinal fasciculus; L, left; MD, mean diffusivity; PCR, posterior corona radiata; PLIC, posterior limb of the internal capsule; PTR, posterior thalamic radiations; RD, radial diffusivity; RLIC, retrolenticular part of internal capsule; SLF, superior longitudinal fasciculus; UF, uncinate fasciculus.

**Table S4. Results of the jackknife analysis in the pooled meta-analysis of 16 TBSS studies.**

| Discarded study | No FA alteration |
| --- | --- |
| Andrade.2019^14^ | Yes |
| Zhou.2018^3^ | Yes |
| Bollettini.2018^6^ | Yes |
| Yagi.2017^13^ | Yes |
| Hawco.2017^7^ | Yes |
| Gan.2017^4^ | Yes |
| Magioncalda.2016^12^ | Yes |
| Ameis.2016^15^ | Yes |
| Fan.2015^11^ | Yes |
| Rosso.2014^18^ | Yes |
| Fitzgerald.2014^16^ | Yes |
| Silk.2013^19^ | Yes |
| Benedetti.2013^5^ | Yes |
| Jayarajan.2012^17^ | Yes |
| Zarei.2011^20^ | No |
| Nakamae.2011^1^ | Yes |

Note: “Yes/No” demonstrate that the “no alterations of FA value” is/is not significant in the jackknife analysis, respectively. FA, fractional anisotropy; TBSS, tract-based spatial statistics.

**Table S5. Results of the jackknife analysis in the subgroup meta-analysis of 10 studies with adult patients with OCD.**

| Discarded study | Decreased FA in the genu and anterior body of corpus callosum |
| --- | --- |
| Andrade.2019^14^ | Yes |
| Zhou.2018^3^ | Yes |
| Bollettini.2017^6^ | Yes |
| Yagi.2017^13^ | Yes |
| Hawco.2017^7^ | Yes |
| Gan.2017^4^ | Yes |
| Magioncalda.2016^12^ | Yes |
| Fan.2015^11^ | Yes |
| Benedetti.2013^5^ | Yes |
| Nakamae.2011^1^ | Yes |

Note: “Yes/No” demonstrate that the FA reduction in the corpus callosum is/is not significant in the jackknife analysis, respectively. FA, fractional anisotropy.

**Figure S1. Results of the funnel plots assessing the publication bias for** **the 10 studies with adult patients with obsessive compulsive disorder (OCD).**


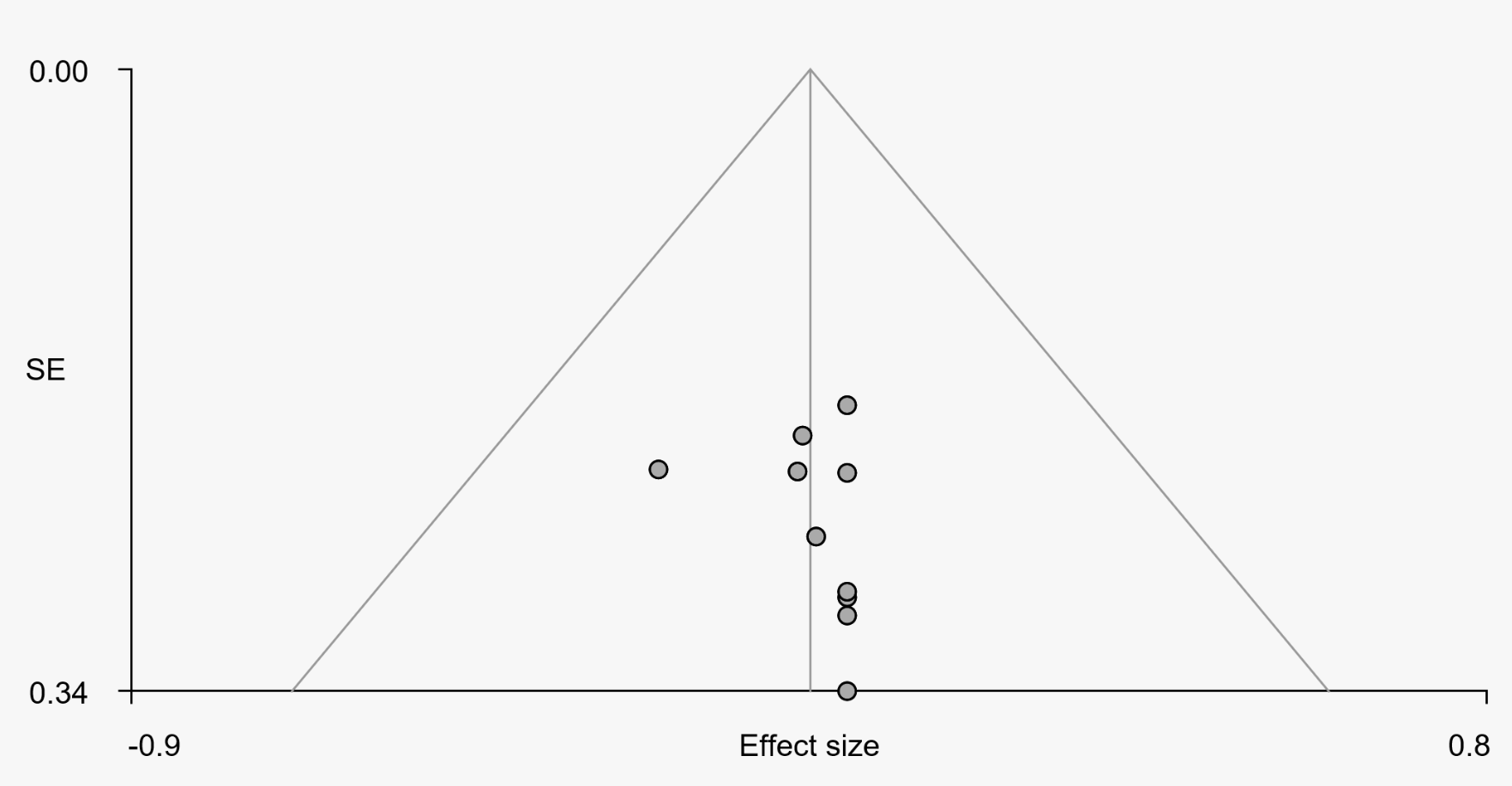


For the 10 TBSS studies with adult patients with OCD, the Egger’s test and funnel plots revealed no significant publication bias in the genu and anterior body of corpus callosum (Bias =0.40, *t* = 0.66, *df* = 8, *P* = 0.528) in the adult patients with OCD.

**References**

1. Nakamae T, et al. Diffusion tensor imaging and tract-based spatial statistics in obsessive-compulsive disorder. *J Psychiatr Res*. *2011*;**45**(5):687-90.

2. Bora E, et al. White matter microstructure in patients with obsessive-compulsive disorder. *J Psychiatry Neurosci*. *2011*;**36**(1):42-6.

3. Zhou C, et al. Cortical thickness and white matter integrity abnormalities in obsessive-compulsive disorder: A combined multimodal surface-based morphometry and tract-based spatial statistics study. *Depress Anxiety*. *2018*;**35**(8):742-51.

4. Gan J, et al. Abnormal white matter structural connectivity in adults with obsessive-compulsive disorder. *Transl Psychiatry*. *2017*;**7**(3):e1062.

5. Benedetti F, et al. Widespread changes of white matter microstructure in obsessive-compulsive disorder: effect of drug status. *Eur Neuropsychopharmacol*. *2013*;**23**(7):581-93.

6. Bollettini I, et al. White matter alterations associate with onset symptom dimension in obsessive-compulsive disorder. *Psychiatry Clin Neurosci*. *2018*;**72**(1):13-27.

7. Hawco C, et al. Age and gender interactions in white matter of schizophrenia and obsessive compulsive disorder compared to non-psychiatric controls: commonalities across disorders. *Brain Imaging Behav*. *2017*;**11**(6):1836-48.

8. Fontenelle LF, et al. White Matter Changes in OCD Revealed by Diffusion Tensor Imaging. *CNS Spectr*. *2011*;**16**(5):101-9.

9. Spalletta G, Piras F, Fagioli S, Caltagirone C, Piras F. Brain microstructural changes and cognitive correlates in patients with pure obsessive compulsive disorder. *Brain Behav*. *2014*;**4**(2):261-77.

10. Hartmann T, Vandborg S, Rosenberg R, Sorensen L, Videbech P. Increased fractional anisotropy in cerebellum in obsessive-compulsive disorder. *Acta Neuropsychiatr*. *2016*;**28**(3):141-8.

11. Fan S, et al. Mild White Matter Changes in Un-medicated Obsessive-Compulsive Disorder Patients and Their Unaffected Siblings. *Front Neurosci*. *2015*;**9**:495.

12. Magioncalda P, Martino M, Ely BA, Inglese M, Stern ER. Microstructural white-matter abnormalities and their relationship with cognitive dysfunction in obsessive-compulsive disorder. *Brain Behav*. *2016*;**6**(3):e00442.

13. Yagi M, et al. Relationship between symptom dimensions and white matter alterations in obsessive-compulsive disorder. *Acta Neuropsychiatr*. *2017*;**29**(3):153-63.

14. Andrade J, et al. An MRI Study of the Metabolic and Structural Abnormalities in Obsessive-Compulsive Disorder. *Front Hum Neurosci*. *2019*;**13**(186).

15. Ameis SH, et al. A Diffusion Tensor Imaging Study in Children With ADHD, Autism Spectrum Disorder, OCD, and Matched Controls: Distinct and Non-Distinct White Matter Disruption and Dimensional Brain-Behavior Relationships. *Am J Psychiatry*. *2016*;**173**(12):1213-22.

16. Fitzgerald KD, Liu Y, Reamer EN, Taylor SF, Welsh RC. Atypical frontal-striatal-thalamic circuit white matter development in pediatric obsessive-compulsive disorder. *J Am Acad Child Adolesc Psychiatry*. *2014*;**53**(11):1225-33, 33.e1-9.

17. Jayarajan RN, et al. White matter abnormalities in children and adolescents with obsessive-compulsive disorder: a diffusion tensor imaging study. *Depress Anxiety*. *2012*;**29**(9):780-8.

18. Rosso IM, et al. Brain white matter integrity and association with age at onset in pediatric obsessive-compulsive disorder. *Biol Mood Anxiety Disord*. *2014*;**4**(1):13.

19. Silk T, Chen J, Seal M, Vance A. White matter abnormalities in pediatric obsessive-compulsive disorder. *Psychiat Res*. *2013*;**213**(2):154-60.

20. Zarei M, et al. Changes in Gray Matter Volume and White Matter Microstructure in Adolescents with Obsessive-Compulsive Disorder. *Biol Psychiat*. *2011*;**70**(11):1083-90.
